# Geometrical prediction of cleavage planes in crystal structures

**DOI:** 10.1107/S2052252521007272

**Published:** 2021-08-20

**Authors:** Uriel Vaknin, Dov Sherman, Semën Gorfman

**Affiliations:** a Department of Materials Science and Engineering, Tel Aviv University, Wolfson Building for Mechanical Engineering, Tel Aviv, 6997801, Israel; b School of Mechanical Engineering, Tel Aviv University, Wolfson Building for Mechanical Engineering, Tel Aviv, 6997801, Israel

**Keywords:** cleavage, transformations, lattice planes, *GALOCS*, computational modelling, inorganic materials, planar gaps

## Abstract

Cleavage is the ability of single crystals to split easily along specifically oriented planes. This phenomenon is of great interest for materials’ scientists. Acquiring the data regarding cleavage is essential for the understanding of brittle fracture, plasticity and strength, as well as for the prevention of catastrophic device failures. Unfortunately, theoretical calculations of cleavage energy are demanding and often unsuitable for high-throughput searches of cleavage planes in arbitrary crystal structures. A simplified geometrical approach (*GALOCS* = gaps locations in crystal structures) is suggested for predicting the most promising cleavage planes. *GALOCS* enumerates all the possible reticular lattice planes and calculates the plane-average electron density as a function of the position of the planes in the unit cell. The assessment of the cleavage ability of the planes is based on the width and depth of planar gaps in crystal structures, which appear when observing the planes lengthwise. The method is demonstrated on two-dimensional graphene and three-dimensional silicon, quartz and LiNbO_3_ structures. A summary of planar gaps in a few more inorganic crystal structures is also presented.

## Abstract 2

A new geometrical approach is proposed for finding the most prominent planar gaps/cleavage planes in crystal structures. The approach is realized by using the *GALOCS* program package and is demonstrated on several inorganic structures.

## 1. Introduction   

Cleavage is the ability of single crystals to split easily along specifically oriented planes (Hurlbut & Klein, 1977 ▸). Such planes are usually parallel to the reticular lattice planes with low Miller indices/large interplanar distances. The cleavage phenomenon was known long before the discovery of X-ray diffraction by crystals; the observation of cleavage in crystals contributed greatly to the ideas about periodicity/long-range order of their structures (Tutton, 1922 ▸; Authier, 2013 ▸). Cleavage is the most striking example of anisotropy of physical properties (Nye, 1985 ▸). Finally, cleavage is the subject of many materials science oriented research (Gilman, 1960 ▸; Lawn, 1974 ▸): acquiring the information concerning cleavage in a given crystalline material is essential for the understanding of failure, brittle fracture, toughness, plasticity and strength (Lawn *et al.*, 1993 ▸).

The prediction and discovery of cleavage planes is important for microelectronics and electro-optics where durability of crystalline materials and prevention of their catastrophic mechanical failures is critical (Spearing, 2000 ▸). Whether performed theoretically or experimentally, such prediction presents a challenging task.

The simpler approach to the prediction of cleavage planes involves counting the number of broken chemical bonds per unit area of a candidate plane. Such counting can be carried out analytically for the simplest crystal structures (*e.g.* of rock salt, diamond or sphalerite type) (Ramaseshan, 1946 ▸). However, it becomes impractical for larger (*e.g.* ternary) structures. Accurate calculations of cleavage energies are demanding (Zhang *et al.*, 2007 ▸; Bitzek *et al.*, 2015 ▸) and should involve *e.g.* density functional theory (DFT) calculation of the surface energies (Ong *et al.*, 2013 ▸; Tran *et al.*, 2016 ▸), which is half of the cleavage energy [for the case of slowly propagating cracks (Griffith, 1921 ▸)].

It is also possible to study cleavage experimentally (Cramer *et al.*, 2000 ▸; Field, 1971 ▸; Jaccodine, 1963 ▸; Lawn *et al.*, 1993 ▸; Michot, 1987 ▸; Sherman *et al.*, 2008 ▸). While some cleavage planes may immediately appear because of a ‘mechanical impact’, such impact ‘experiments’ can hardly be helpful in the precise measurement of a cleavage energy. More complex arrangements are implemented for this purpose (Gilman, 1960 ▸; Gleizer & Sherman, 2014 ▸; Sherman & Gleizer, 2014 ▸; Hirsh *et al.*, 2020 ▸). Specifically, a single crystal should be cut to a plate whose normal is parallel to a candidate cleavage plane while a uniaxial stress (so-called Mode I stress) must be applied in a controlled manner. In conclusion, existing experimental and theoretical methods are demanding and unsuitable for throughput studies of cleavage without preliminary suggestions of several likely possibilities.

Here, we suggest a simple geometrical algorithm and a computer program *GALOCS* (gaps’ locations in crystal structures) which searches for promising cases of cleavage in single crystals of known structures. Apart from accepting the working hypothesis about the planar character of cleavage, we assume that such cleavage occurs along the planes, exposing the widest planar gaps (the intervals of empty space, appearing when observing the plane lengthwise). The manual variant of this approach is common in crystallography classes, where a lecturer exposes a three-dimensional structural model and demonstrates prominent clearances/gaps in the structure to the audience. While such inspection can be performed both physically and graphically [*e.g.* by using the *VESTA* program (Momma & Izumi, 2011 ▸)], there are no computational methods for doing it automatically. This methodological gap is filled by the *GALOCS* package. In addition to its great illustrative potential, *GALOCS* can serve as a rough predictor of cleavage planes in crystals. If necessary, such planes can be analysed rigorously using DFT. Alternatively, the results may suggest a particular experimental geometry that allows measuring the cleavage energy of the plane.

## 2. The algorithm behind *GALOCS*   

The proposed approach realizes an automatic (as opposed to visual) inspection of the ‘space-filling function’ (SFF). Specifically, we suggest inspecting the sections of the SFF by the planes that are parallel to the lattice planes with reasonable Miller indices/interplanar distances. The dependence of the average SFF on the depth of the plane in the unit cell is produced in order to locate the most prominent planar gaps and measure their width and depth. The correlation between the width/depth of the gap and the cleavage ability of the candidate plane is the major hypothesis behind the algorithm.

### 2.1. Space-filling function   

Calculation of the SFF requires knowledge of space group type, lattice parameters, coordinates, occupancies of atomic/Wyckoff positions and types of atoms. The first definition of the SFF is the superposition of electron densities of individual pseudo-atoms:
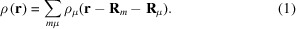
Here, **R**_*m*_ = *u*_*mi*_ **a**_*i*_ (Einstein summation over the repeated index running from one to three is implemented everywhere throughout the article) are the positions of all the lattice nodes, *u*_*mi*_ are arbitrary integers and **a**_*i*_ are the lattice basis vectors *i* = 1, 2, 3. The vectors 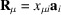 (0 ≤ *x*_μ*i*_ < 1) list the positions of all the atoms in a unit cell. 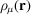 are electron densities of a (pseudo)atom number μ, which can be approximated using *e.g.* electron density of an isolated atom (Su & Coppens, 1998 ▸) or atomic invarioms (Dittrich *et al.*, 2004 ▸). All the atomic electron densities are normalized: 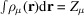 [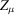 is the number of electrons, associated with the (pseudo)atom number μ].

Another possible definition of the SFF involves an atomic probability density function (PDF). It describes the probability of finding an atom μ displaced by a vector **u** from its average position **R**_μ_. Such displacements may originate from either a thermal motion or a static disorder. A PDF corresponds to the average of all the atomic positions over time or over different unit cells. A three-dimensional Gaussian function is the simplest approximation of an atomic PDF (Coppens, 1997 ▸):
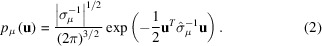
Here, 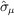 is the second-rank tensor of atomic displacement. The components of this tensor are known from an X-ray or neutron diffraction experiment and are represented as isotropic or anisotropic atomic displacement parameters (*U*_*ij*_ or β_*ij*_) (Trueblood *et al.*, 1996 ▸; Coppens, 1997 ▸; Tsirelson & Ozerov, 1996 ▸). If 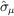 is represented by a 3 × 3 matrix then **u** ^*T*^ and **u** are the rows or the column vectors, respectively. The SFF ρ(**r**) can now be calculated as
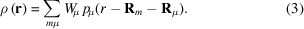
Here, 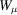 describe the ‘weights’ of every atom in the cumulative SFF. 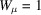 yields an SFF in the form of atomic density (it disregards the sort of atoms involved). Setting 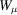 to the atomic masses defines the SFF as the mass density. Finally, setting 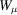 to 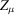 will produce an SFF in the form of nuclei charge density.

Notably, all possible definitions of the density function sustain the periodicity so that ρ(**r**) = ρ(**r** + **R**_*m*_). Therefore, it is sufficient to calculate the values of ρ(**r**) inside the unit cell only. Because both 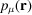 and 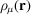 are negligible when |**r**| > *r*_max_ (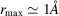), only the limited number of atoms whose centres are inside the unit cell or within *r*_max_ from its borders are included in the sums (1) or (3).

### 2.2. Forming a section of a unit cell by an arbitrary candidate cleavage plane   

**Figure 1:**
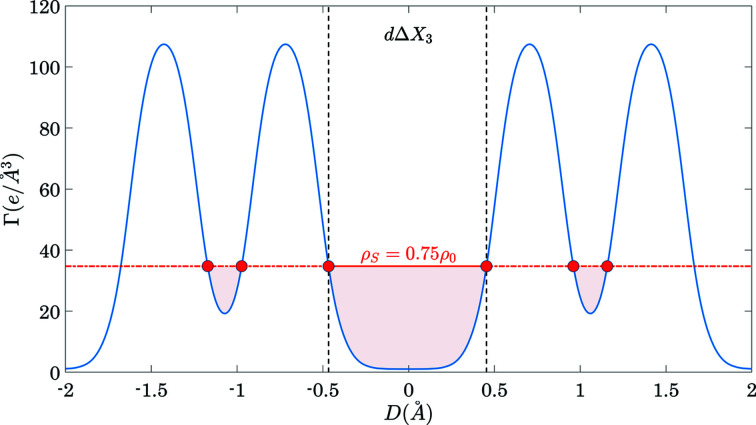
A graphical illustration of the definition of *C*_*hkl*_ according to equation (7). The solid line shows an arbitrary Γ(*hkl*, *D*) function extending over two unit cells (one unit cell on the positive and one unit cell on the negative side of the *D* axis). The dashed horizontal line shows the threshold value of ρ_*S*_ = 0.75ρ_0_. The regions of Γ(*hkl*, *D*) < ρ_*S*_ are filled by colour. The numerator of equation (7) is equal to the area inside the coloured region.

*GALOCS* inspects the sections of the SFF by the planes that are perpendicular to a unitary vector **n** (|**n**| = 1). Let us define the depth *D* of the plane inside the crystal and introduce the orientation and depth-dependent average Γ(**n**, *D*) of the SFF ρ(**r**) as
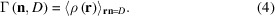
Here the averaging of ρ(**r**) is performed over all the positions **r**, satisfying the condition **rn** = *D* (the equation of a plane, normal to the vector **n** and standing at the distance *D* from the origin). The periodicity of the crystal structure means that *D* is non-negative and below the corresponding interplanar distance *d*(**n**) = *d*_*hkl*_. The latter is the inverse length of the primitive reciprocal lattice vector, 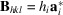 {*h*_*i*_ or *h*, *k*, *l* are coprime integers/Miller indices of the plane [the Miller indices are coprime if a primitive unit cell is used (Nespolo, 2015 ▸)] and 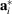 are the basis vectors of the reciprocal lattice 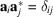} where **B**_*hkl*_ ∥ **n**: 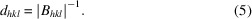
It is sufficient to average the SFF ρ(**r**)_**rn** = *D*_ over translationally independent locations only, rather than over the entire plane. It is therefore worth transforming the coordinates of **r** to the coordinate system **A**_1_, **A**_2_, **A**_3_ such that **A**_*i*_ and **a**_*i*_ are the bases of the same crystal lattice, but the vectors **A**_1_ and **A**_2_ are parallel to the plane of interest (*hkl*) and **A**_3_ connects two adjacent lattice planes (*A*_3*i*_ *h*_*i*_ = 1). The number-theoretical algorithms for such a transformation are described elsewhere (Gorfman, 2020 ▸) and the corresponding MATLAB-based program *MULDIN* is deposited there. This step presents the core of the algorithm because it uses the periodicity of the crystal structure. Using the coordinates *X*_1_, *X*_2_ and *X*_3_ such that **r** = *X*_*i*_ **A**_*i*_ = *x*_*i*_ **a**_*i*_ and expressing the SFF ρ(*x*_1_, *x*_2_, *x*_3_) as ρ_*hkl*_(*X*_1_, *X*_2_, *X*_3_) yields
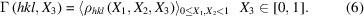
The cleavage planes are likely to appear where Γ(*hkl*, *X*_3_) drops to the lowest possible values. Averaging of Γ(*hkl*, *X*_3_) over *X*_3_ would yield the average of the SFF 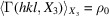 over the entire unit cell. Let us introduce the constant threshold ρ_*S*_ = *y*ρ_0_ (typically *y* = 0.75) such that all the *X*_3_ values where Γ(*hkl*, *X*_3_) < ρ_*S*_ are considered as planar gaps. We define the maximum effective width of a planar gap as
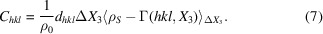
Here Δ*X*_3_ is the length of the longest continuous range of *X*_3_ values where Γ(*hkl*, *X*_3_) < ρ_*S*_ and 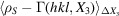 is the average gap depth in this range. The denominator ρ_0_ is introduced in order to reduce the dimension of *C*_*hkl*_ to Å. Fig. 1 ▸ explains definition (7) graphically. It shows an arbitrary Γ(*hkl*, *D*) with the most prominent gap between two vertical dashed lines.

Definition (7) suggests that *C*_*hkl*_ must be centrosymmetric. Indeed, the sets of Miller indices (*hkl*) and 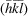 define the same lattice planes. The only difference between them concerns the direction of the plane movement with the increasing value of *X*_3_. Specifically, 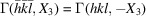, meaning that 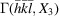 has the same minima, the same average value and the same width of the structural gap as Γ(*hkl*, *X*_3_). Accordingly, 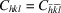, so the symmetry of the *C*_*hkl*_ is defined by one of the eleven Laue classes.

### 2.3. The flow chart of the *GALOCS* algorithm   

*GALOCS* realizes the following steps:

(i) Calculating electron density ρ(*x*_1_, *x*_2_, *x*_3_) (or any other SFF in future releases) inside a unit cell (0 ≤ *x*_*i*_ < 1) as a superposition of spherical atoms.

(ii) Generating the set of symmetry-independent (with respect to the relevant Laue class) lattice planes (*hkl*) such that *d*_*hkl*_ > *d*_min_.

(iii) For each (*hkl*) transforming the basis vectors **a**_*i*_ → **A**_*i*_ using the *MULDIN* algorithm (Gorfman, 2020 ▸), and expressing ρ(*x*_1_, *x*_2_, *x*_3_) as ρ_*hkl*_(*X*_1_, *X*_2_, *X*_3_) (0 ≤ *X*_*i*_ < 1).

(iv) Calculating *C*_*hkl*_ according to equation (7), and drawing the directional dependence of *C*_*hkl*_ on the stereographic projections and polar plots.

The MATLAB-based software package includes several modules for:

(*a*) Automatic reading of relevant structural information from CIFs.

(*b*) Calculating ρ(*x*_1_, *x*_2_, *x*_3_) in the unit cell on the predefined grid. The current version of the program uses pre-tabulated spherically symmetrical electron densities in isolated atoms (Su & Coppens, 1998 ▸).

(*c*) Drawing Γ(*hkl*, *D*) curves for any chosen *hkl* values.

(*d*) Calculation of *C*_*hkl*_ (according to equation 7 with user-defined clearance threshold, typically *y* = 0.75) for the set of lattice planes with interplanar distance above some user-defined value *d*_min_.

The software package of *GALOCS* with corresponding manual is available in the supporting information and through the GitHUB platform. The functionality of the package will be extended (*e.g.* by introduction to the graphical user interface and by implementing various SFF models) subject to the interest of the user community.

### 2.4. Two-dimensional illustration   

**Figure 2:**
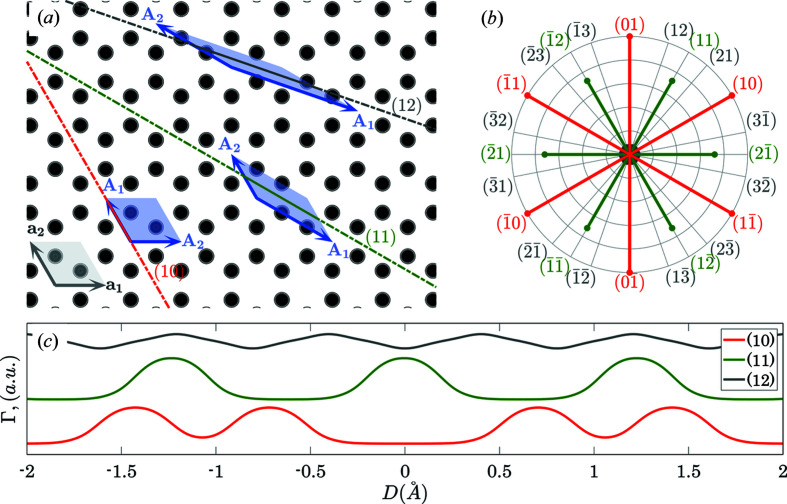
A two-dimensional illustration of the *GALOCS* output for the case of graphene. (*a*) The SFF for the structure of graphene according to equation (1) and electron densities of isolated carbon atoms. The same figure shows the standard unit cell and its three transformations, suitable for the analysis of the corresponding planar gaps in this structure. In all cases, the transformed unit cell has the vector **A**_1_ parallel to the (*hk*) planes. (*b*) The dependence of the *C*_*hk*_ on the direction **n** ∥ **B**_*hk*_. The Miller indices corresponding to each of the directions are marked explicitly. (*c*) The dependences of Γ(10, *X*_3_), Γ(11, *X*_3_) and Γ(12, *X*_3_) on *D* = *d*_*hk*_ *X*_3_ (vertically shifted relatively to each other for clarity).

Fig. 2 ▸ illustrates the *GALOCS* output for a two-dimensional graphene structure. The conventional unit cell is based on the vectors **a**_1_ and **a**_2_ such that 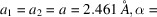 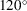. The SFF was defined by the superposition of electron densities of isolated carbon atoms. Fig. 2 ▸(*a*) shows the transformed unit cells [based on the vectors **A**_1_ and **A**_2_ with **A**_1_ being parallel to (*hk*)] for the cases of (10), (11) and (12) planes. Fig. 2 ▸(*b*) illustrates the dependence of *C*_*hk*_ on the reciprocal-space directions using the polar plot. The spikes extend in the directions of the reciprocal lattice vectors **B**_*hk*_, their lengths are proportional to the corresponding *C*_*hk*_ values. It shows that the longest gap in graphene is present for {10}_H_ and {11}_H_ families of planes [the notation 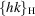 stands for set of planes that are symmetry equivalent to (*hk*) with respect to the operations of the crystal class of two-dimensional hexagonal lattice]. {21}_H_ planes have ∼15 times smaller *C*_*hk*_ (see Table 1 ▸ for the numerical values).

## 3. Examples   

Here, we demonstrate the implementation of the algorithm for the case of three inorganic structures (Si, LiNbO_3_ and SiO_2_).

### 3.1. Silicon/diamond structural type   

**Figure 3:**
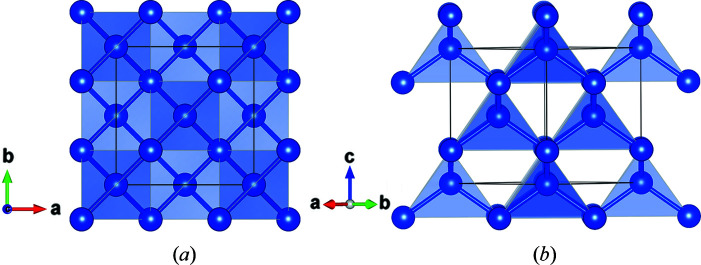
The structure of silicon viewed along (*a*) [001] and (*b*) [110] crystallographic directions. The images were produced using the *VESTA* program (Momma & Izumi, 2011 ▸).

Fig. 3 ▸ projects the structure of silicon along the crystallographic [001] and [110] directions. The structure has one symmetry-independent atom sitting at the standard origin 1 of the space group 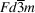. Each atom has four nearest neighbours at the corners of a regular tetrahedron. Table 2 ▸ summarizes the relevant structural information.

**Figure 4:**
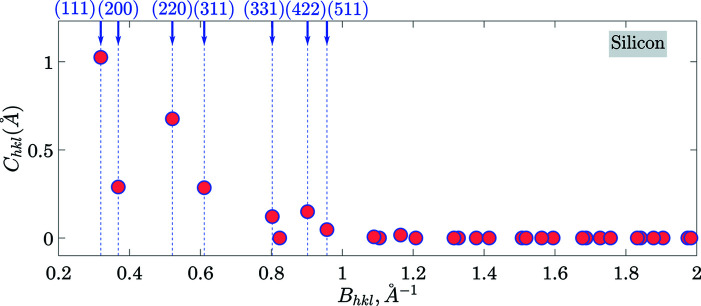
The *B*_*hkl*_ dependence of *C*_*hkl*_ values in silicon. Here *B*_*hkl*_ is the length of the corresponding reciprocal **lattice vector (inverse to *d*_*hkl*_)**. The Miller indices are given with respect to the non-primitive (face centred) unit cell; accordingly, some Miller indices [*e.g.* (220)] have a common divider. When converted to the primitive unit cell, these indices are coprime.

The calculation of *C*_*hkl*_ over the lattice planes with the interplanar distance above 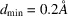 involved 17 330 primitive reciprocal lattice vectors enclosed in a corresponding reciprocal-space sphere of radius 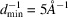. This set included 446 symmetry-independent planes (with respect to the operations of the point-symmetry group 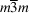). The cut-off *d*_min_ is justified by the fact that *C*_*hkl*_ drops to zero for those planes that have small interplanar distances. Fig. 4 ▸ illustrates this statement by showing *C*_*hkl*_ as a function of the length of the primitive reciprocal lattice vector (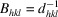). Prominent gaps are present among low *B*_*hkl*_ (high *d*_*hkl*_) planes only. The same figure indicates the Miller indices of the planes with the highest cleavage ability. Note that the indices of the planes are given with respect to the conventional non-primitive (face centred) unit cell and therefore some of the indices are not coprime. The indices are coprime if expressed using a primitive unit-cell basis (Nespolo, 2015 ▸).

**Figure 5:**
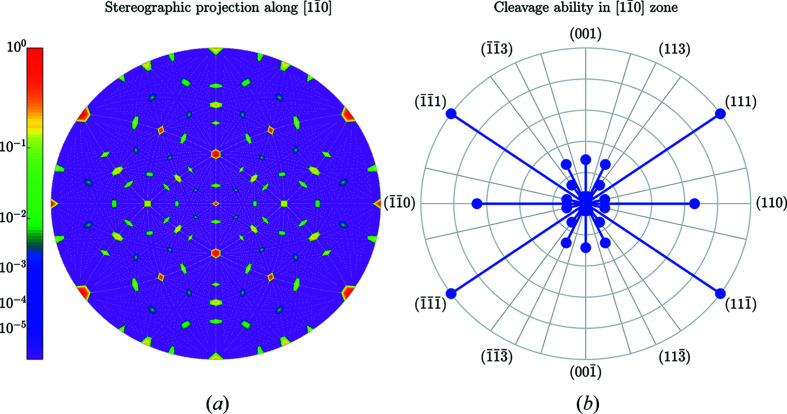
Anisotropy of *C*_*hkl*_ values in silicon. (*a*) The stereographic projection viewed along the 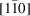 crystallographic direction displays the false-colour map of the *C*_*hkl*_ (dependence of *C*_*hkl*_ on the direction). Each point on the stereographic projection corresponds to a specific reciprocal-space direction. The small white points indicate the stereographic projections of the normals to all the lattice planes involved in the calculation. The colour scheme expresses the values 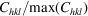 according to the colour bar on the left. (*b*) The polar diagram of the cleavage ability for all the planes in the 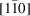 zone. The outer circle corresponds to the highest cleavage ability (*C*_111_ = 1.025 Å for the case of silicon).

Figs. 5 ▸ and 6 ▸ illustrate the striking anisotropy of *C*_*hkl*_ in silicon using stereographic projections and polar plots. The stereographic projections contain a false-colour map, which sets one-to-one correspondence between the colour and the *C*_*hkl*_ values. The projection is viewed along the zone axis **A**_*uvw*_ = [*uvw*] = *u* **a**_1_ + *v* **a**_2_ + *w* **a**_3_ {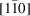/[111] in Figs. 5 ▸(*a*)/6 ▸(*a*), respectively}. Figs. 5 ▸(*b*) and 6 ▸(*b*) show polar plots of *C*_*hkl*_ for all the reciprocal lattice directions **B**_*hkl*_ in the zone (*i.e.* such that **B**_*hkl*_ · **A**_*uvw*_ = 0). The individual spikes extend in the directions that are normal to the anticipated cleavage planes. Such polar plots provide specific guidelines for the cleavage experiment, in which a crystal is prepared in the form of a wafer whose surface is normal to **A**_*uvw*_. Cleavage is initiated at a small pre-crack [see *e.g.* (Hirsh *et al.*, 2020 ▸)] and at a uniaxial stress [also known as Mode I stress (Lawn *et al.*, 1993 ▸)] along the direction **n**. Table 3 ▸ lists numerical values for the cleavage ability of the most prominent planes.

The calculations predict {111}_C_ and {110}_C_ as the most prominent cleavage planes in silicon [the notation 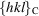 stands for the set of planes that are symmetry equivalent to 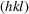 with respect to the operations of the cubic crystal class 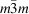]. These planes are well known from real cleavage experiments on single crystals of silicon (Gleizer *et al.*, 2014 ▸).

**Figure 7:**
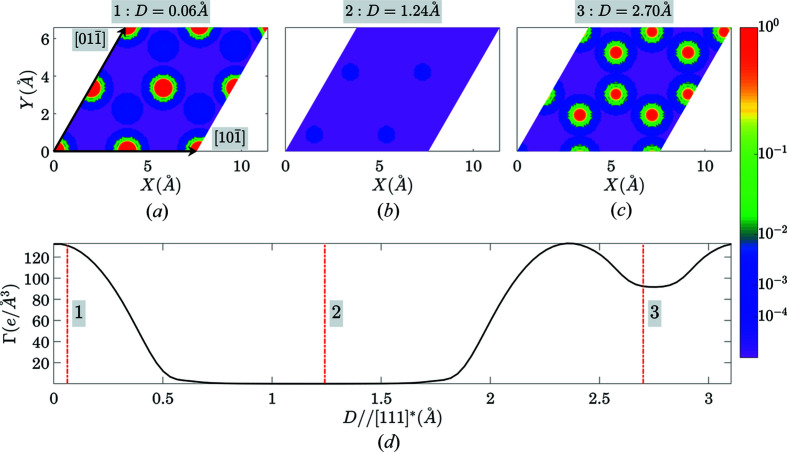
An illustration of the structural-gap location for the case of the (111) plane in silicon. The parts (*a*)–(*c*) show three electron-density sections by the (111) plane at three various positions of the plane in the unit cell, while (*d*) shows the Γ(111, *D*) function with the vertical lines at the positions corresponding to the electron-density sections in (*a*)–(*c*).

**Figure 8:**
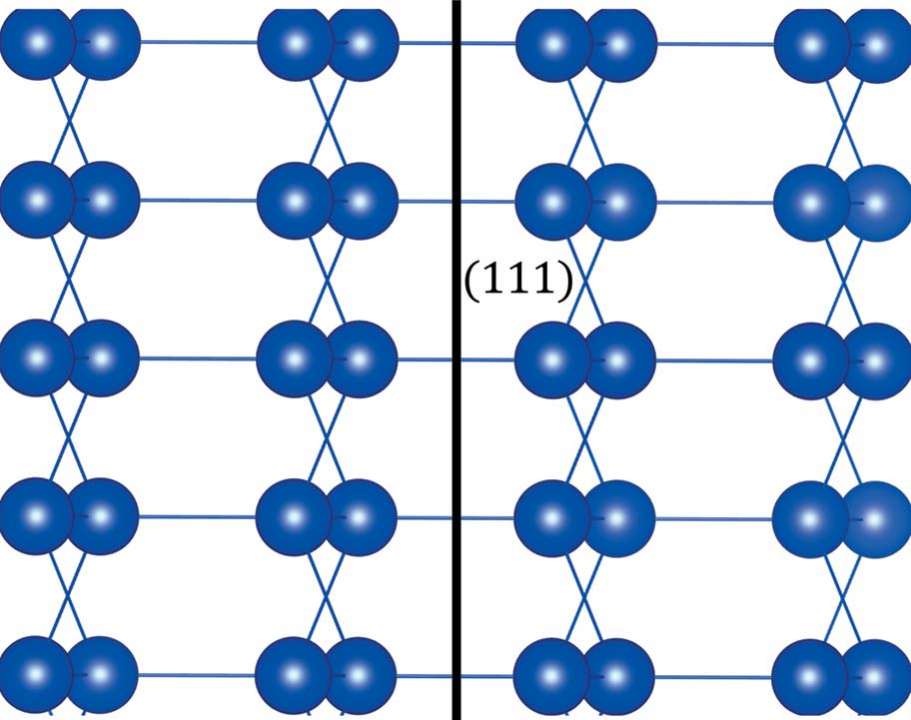
The structure of silicon viewed along the (111) plane. The [111]* reciprocal lattice direction is horizontal. The figure exposes the corresponding structural gap (as also appears in Fig. 7 ▸).

The output is further illustrated in Figs. 7 ▸ and 8 ▸. Fig. 7 ▸ shows the corresponding sections of electron density by (111) planes and the Γ(**n**, *D*) function where the gap is seen clearly. Fig. 8 ▸ shows the view of the structure along the predicted cleavage plane. This figure was produced using the *VESTA* program, where (111) plane was added artificially and the structural model was rotated in a way that the normal to the plane is parallel to the horizontal axis of the screen. The easy ability to produce the images, exposing the structural gaps in the crystal clearly, is one of the goals of the suggested algorithm.

### 3.2. Quartz (SiO_2_)   

**Figure 9:**
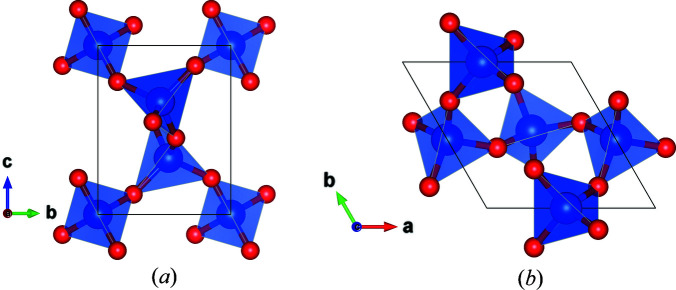
The structure of quartz viewed along [100] and [001] crystallographic directions. The images were produced using *VESTA* (Momma & Izumi, 2011 ▸).

**Figure 10:**
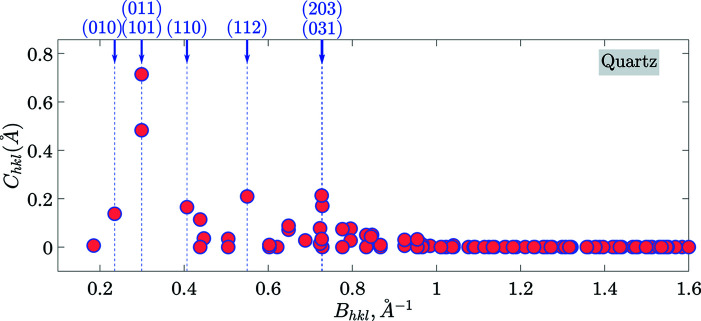
Same as Fig. 4 ▸ but for the case of quartz.

**Figure 11:**
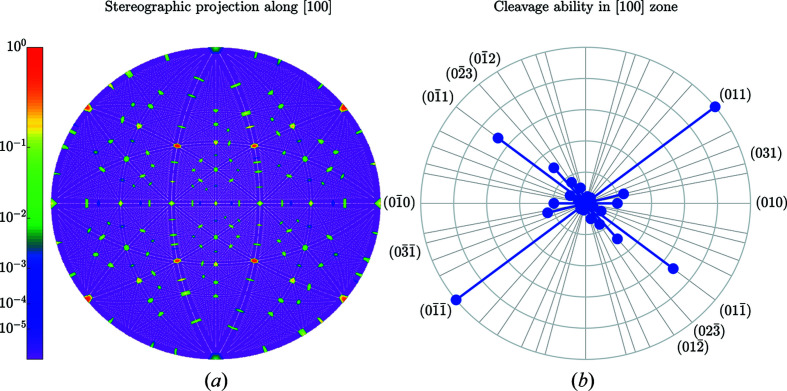
Same as Fig. 5 ▸ but for the [100] direction in quartz. The outer circle in (*b*) corresponds to the highest cleavage ability *C*_011_ = 0.714 Å.

**Figure 13:**
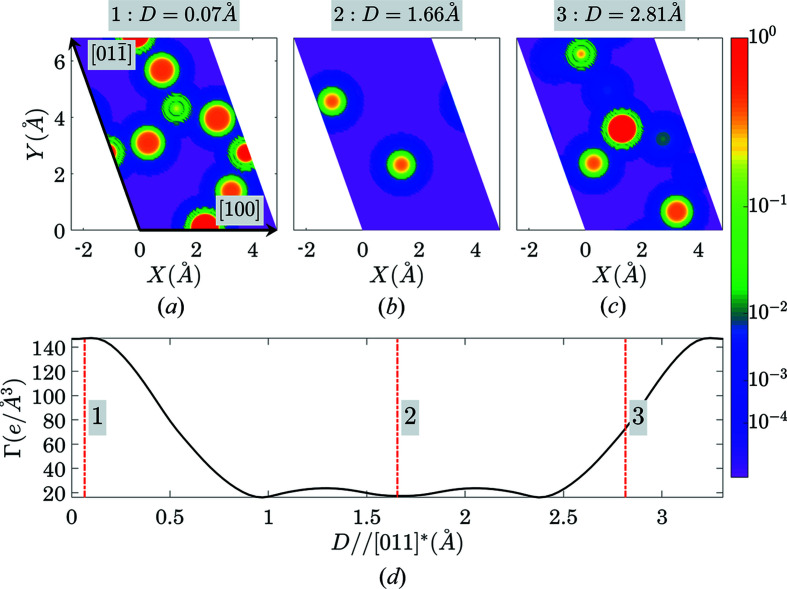
An illustration of the gap location for the case of the (011) plane in quartz. Parts (*a*)–(*c*) show three electron-density sections by the (011) plane at three various positions of the plane in the unit cell, while (*d*) shows the Γ(011, *D*) function with the vertical lines at the positions corresponding to the electron-density sections in (*a*)–(*c*).

Fig. 9 ▸ projects the structure of quartz along [100] and [001] directions, while Table 4 ▸ summarizes the relevant structural information (Levien *et al.*, 1980 ▸). Right-handed quartz crystallizes in the space group type *P*3_2_21 (No. 154) and corresponds to the Laue class 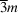. The structure has two symmetry-independent atoms (Si and O). Enumeration of the lattice planes with the interplanar distance above 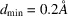 results in 49 358 reciprocal lattice vectors, 4393 of them are symmetry independent (with respect to the point-symmetry operations of the Laue class). Figs. 10 ▸–12 ▸ ▸ and Table 5 ▸ show the results of the calculations (as in chapter 3.1). Fig. 10 ▸ illustrates the dependence of *C*_*hkl*_ on the length of the reciprocal lattice vectors. Figs. 11 ▸ and 12 ▸ illustrate the anisotropy of *C*_*hkl*_ using stereographic projections along [100] and [001] zone axes [defining the normal to *X* and *Z* cuts of quartz wafers (Brainerd *et al.*, 1949 ▸)]. Table 5 ▸ lists the numerical values of *C*_*hkl*_ of seven planes with the most prominent gaps. Figs. 13 ▸ and 14 ▸ (organized in the same way as Figs. 7 ▸ and 8 ▸) illustrate the output for the case of (011) planes in quartz.

### 3.3. Lithium niobate (LiNbO_3_)   

LiNbO_3_ crystallizes in the space group type *R*3*c* (Weis & Gaylord, 1985 ▸; Weigel *et al.*, 2020 ▸), Laue class 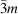. Table 6 ▸ summarizes the relevant structural information. The structure has three symmetry-independent atoms (Li, Nb and O).

**Figure 15:**
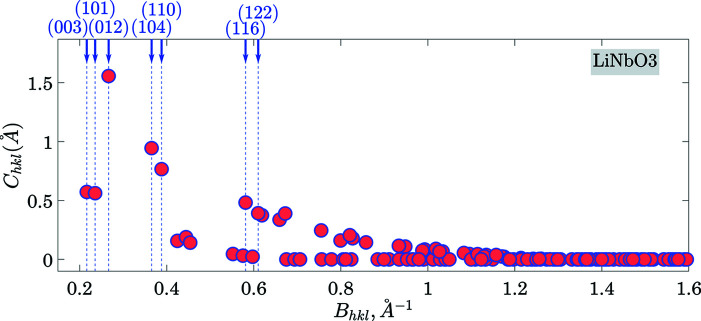
Same as Fig. 4 ▸ but for the case of LiNbO_3_.

Enumeration of the lattice planes with the interplanar distance exceeding 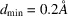 results in the generation of 46 340 reciprocal lattice vectors, where 4108 of them are symmetry independent (with respect to the point-symmetry group 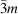). The calculation results are presented in Figs. 15 ▸–19 ▸ ▸ ▸ ▸ and Table 7 ▸, organized as in the previous sections. The analysis predicts the most prominent cleavage plane with the Miller indices (012). This plane is also known from the measurements of cleavage energies in LiNbO_3_ crystals (Hirsh *et al.*, 2020 ▸).

## 4. Further examples   

Tables 8 ▸–12 ▸ ▸ ▸ ▸ present a brief summary of the *C*_*hkl*_ calculations for the structures of wurzite (AlN), fluorite (CaF_2_), diamond (C), pyrite (FeS_2_) and corundum (Al_2_O_3_). They are organized in the same way as Tables 3 ▸, 5 ▸ and 7 ▸. All the examples are included in the *GALOCS* user manual in the supporting information.

## 5. Discussion   

*GALOCS* is an easy and illustrative way to find planar gaps in arbitrary crystal structures. Although we do not claim a one-to-one correspondence between the size of these gaps and the cleavage energies, some correlation between them exists. Specifically, the calculations in Section 3.1 suggest that the most prominent gap is seen along (111) and the next most prominent is seen along (110) planes. This result is proven by both experiments (Gleizer *et al.*, 2014 ▸) and calculations (Pérez & Gumbsch, 2000 ▸), suggesting that single crystals of silicon will indeed break most readily along (111) and next most readily along (110) planes. Specifically, these literature results imply that cleavage along (110) planes requires ∼20% more energy input than cleavage along (111) planes. Table 13 ▸ shows the numerical values of cleavage energies against the calculated parameters of the gaps.

Cleavage of quartz single crystals is debated in the literature [see *e.g.* (White, 2006 ▸; Bloss & Gibbs, 1963 ▸)]. We are unaware of any accurate calculations or precise measurements of the cleavage energies in quartz. Nonetheless, according to Bloss & Gibbs (1963 ▸), when crushed, quartz cleaves most readily along (101)/(011) planes and next most readily along (112) planes. All these planes appear at the top of the list calculated by our algorithm.

Cleavage of LiNbO_3_ crystals was recently investigated by Hirsh *et al.* (2020 ▸), featuring (012), (010) and (116) cleavage planes [the cleavage energies of (012) and (010) were measured]. While all these planes appear on the most prominent planar-gap list in Table 7 ▸, there is some disagreement between the measured cleavage energy and the gap width. Specifically (see Table 14 ▸), while the measured cleavage energy of (010) is ∼40% smaller than that of (012), the gap of (010) is four times narrower than (012). It is important to reiterate that this type of disagreement is expected, while the presence of these planes on the list is the simplified *GALOCS* scheme main goal (since most of the planes do not exhibit any gaps at all).

**Figure 20:**
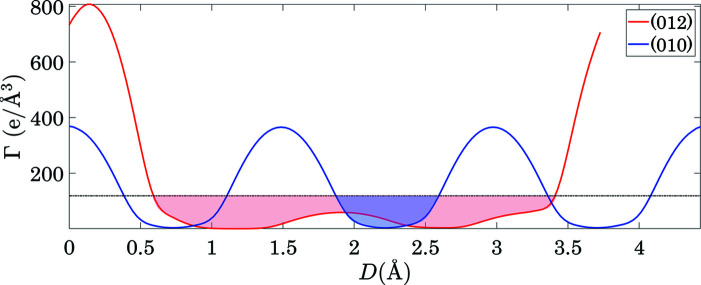
Γ(012, *D*) and Γ(010, *D*) curves for LiNbO_3_. The horizontal dashed line is drawn at the chosen threshold level of 0.75ρ_0_.

Still, in order to investigate the matter deeper, we inspected the Γ(*hkl*, *D*) for both (012) and (010) planes. Fig. 20 ▸ shows that the Γ(012, *D*) curve has a well, which separates the gap into two ‘valleys’. This suggests that the chosen threshold value *y* = 0.75 results in overestimation of the actual gap size for (012) planes. Such close inspection of Γ(*hkl*, *D*) is therefore recommended for all planes that are selected as candidate cleavage planes, and can be carried out by using the *GALOCS* package. Additionally, it is possible to recalculate all the *C*_*hkl*_ values using different *y* thresholds if necessary.

The algorithm can be improved by adding dummy atoms into the structures (*e.g.* to mimic chemical bonds). Alternatively, it may implement advanced models of electron density, which, in turn, is obtained by DFT calculations or results from a multipole model refinement of X-ray diffraction intensities (Hansen & Coppens, 1978 ▸). Additionally, atomic electron densities may be convoluted with their PDFs; this can be particularly valuable for disordered materials with high atomic displacement parameters. In general, the SFF ρ(*x*_1_, *x*_2_, *x*_3_) can be customized, *e.g.* by adding ‘sticks’ (additional electron densities) along the bond lines and removing the atoms themselves. This way the algorithm and the program will be capable of counting the number of chemical bonds intersected by the planes.

## 6. Conclusions   

We developed geometrical algorithm *GALOCS* to locate the planes that expose the most prominent planar gaps in crystal structures. Such planes are listed as candidate cleavage planes, to be explored experimentally or by using density-functional-theory calculations. *GALOCS* implements some known generalized space-filling function (*e.g.* superposition of atomic electron densities or the probability density function). It calculates the average values of this function within specific planes and as a function of plane depth with respect to the unit-cell origin. We also provided detailed illustration of the algorithm for silicon, quartz and LiNbO_3_ where a clear correlation between our calculations and existing experimental studies of cleavage energy is present.

## Supplementary Material

GALOCS package. DOI: 10.1107/S2052252521007272/lt5039sup1.zip

User manual for the GALOCS package. DOI: 10.1107/S2052252521007272/lt5039sup2.pdf

## Figures and Tables

**Figure 6:**
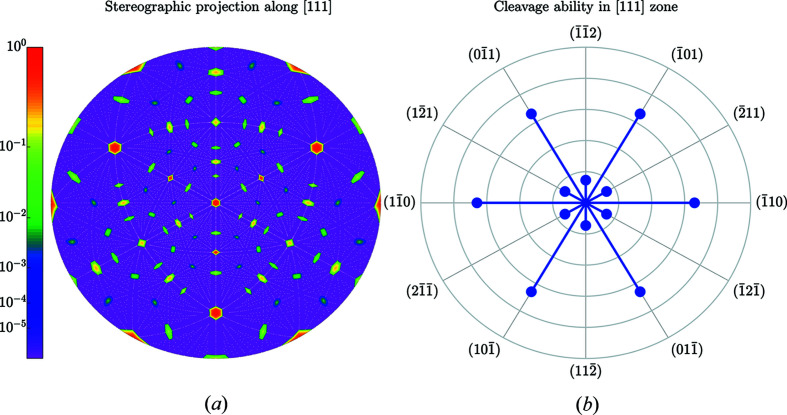
Same as Fig. 5 ▸ but for the [111] direction in silicon. The outer circle in (*b*) corresponds to the highest cleavage ability *C*_111_ = 1.025 Å.

**Figure 12:**
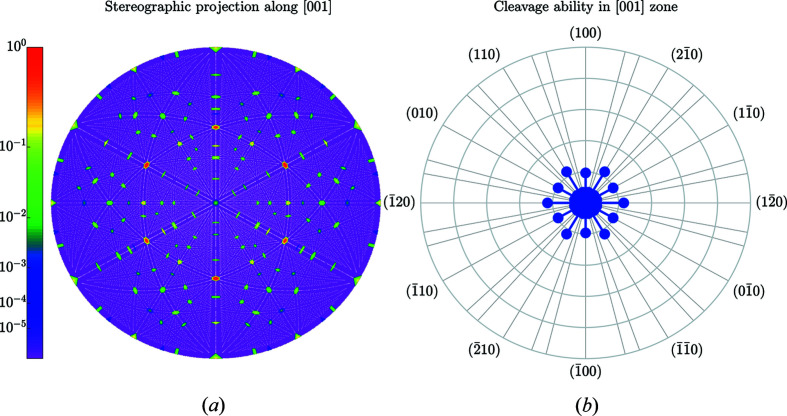
Same as Fig. 5 ▸ but for the [001] direction in quartz. The outer circle in (*b*) corresponds to the highest cleavage ability *C*_011_ = 0.714 Å.

**Figure 14:**
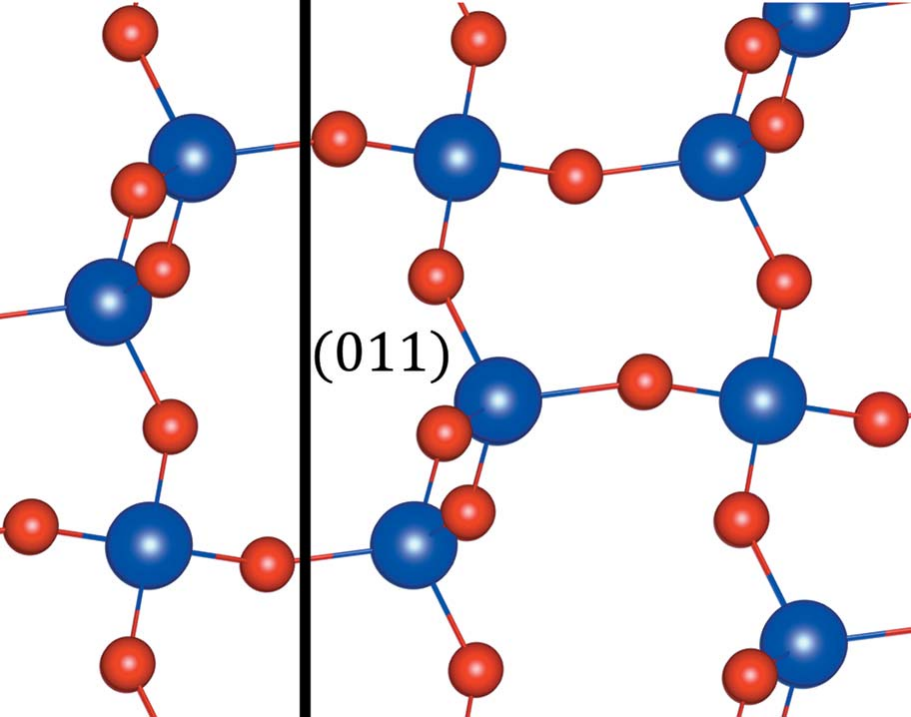
The structure of quartz viewed along the (011) plane. The [011]* reciprocal lattice direction is horizontal.

**Figure 16:**
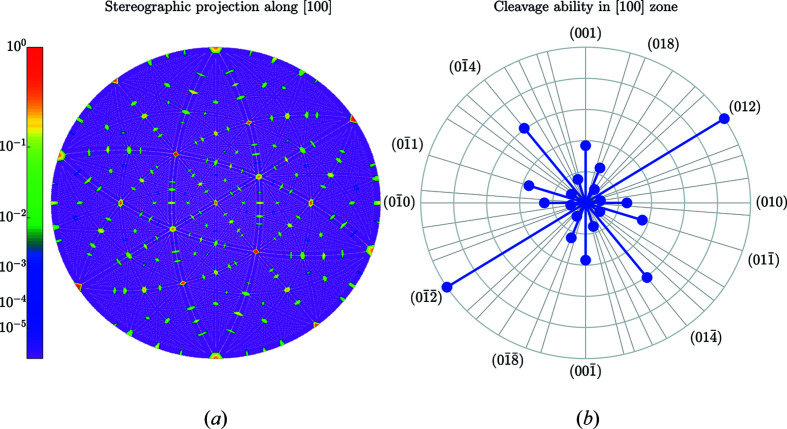
Same as Fig. 5 ▸ but for the [100] direction in LiNbO_3_. The outer circle in (*b*) corresponds to the highest cleavage ability *C*_012_ = 1.556 Å.

**Figure 17:**
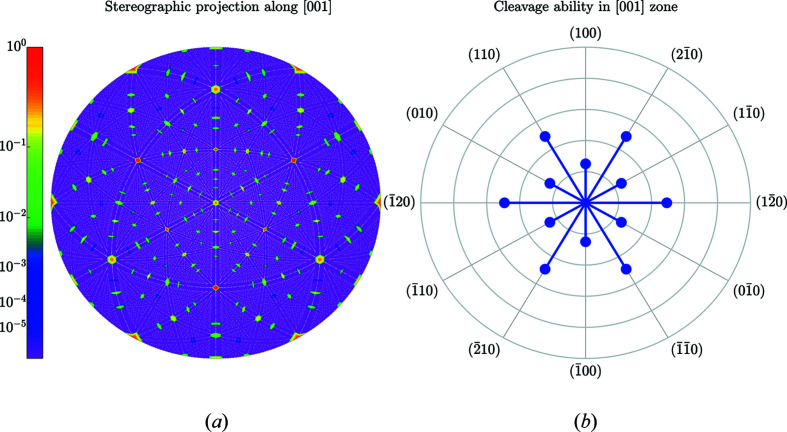
Same as Fig. 5 ▸ but for the [001] direction in LiNbO_3_. The outer circle in (*b*) corresponds to the highest cleavage ability *C*_012_ = 1.556 Å.

**Figure 18:**
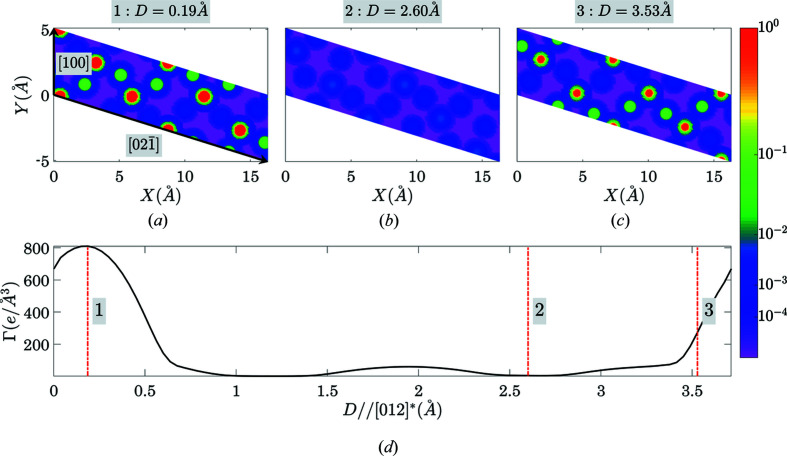
An illustration of the gap location for the case of the (012) plane in LiNbO_3_. Parts (*a*)–(*c*) show three electron-density sections by the (012) plane at three various positions of the plane in the unit cell, while (*d*) shows the Γ(012, *D*) function with the vertical lines at the positions corresponding to the electron-density sections in (*a*)–(*c*).

**Figure 19:**
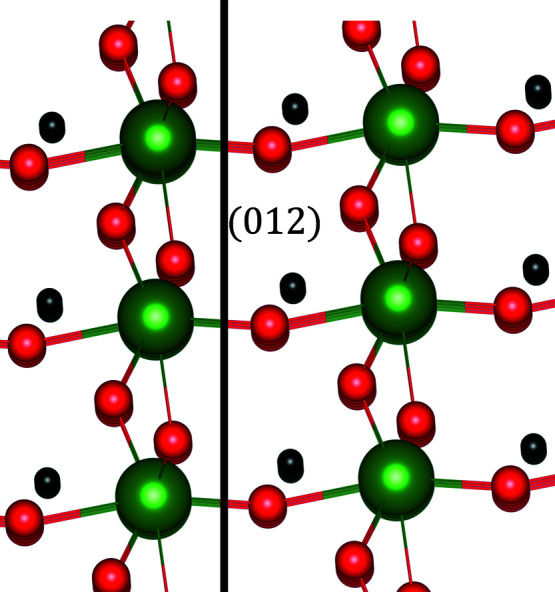
The structure of LiNbO_3_ viewed along the (012) plane. The [012]* reciprocal lattice direction is horizontal.

**Table 1:** Magnitudes of *C*_*hk*_ in graphene

(*hk*)	(10)	(11)	(12)
{C}_{hk} \,(\AA)	0.54	0.40	0.03

**Table:** 

Space group	Fd\bar 3m (No. 227)
Laue class	m\bar 3m
Number of atoms per unit cell	8
Lattice parameter(s) (Å)	a = 5.43

**Table:** Asymmetric unit

Atom name	Charge	Position	Wyckoff letter and multiplicity	Local symmetry
Si	14	[000]	8*a*	\bar 43m

**Table 3:** Magnitudes of *C*_*hkl*_ in silicon

(*hkl*)	(111)	(110)	(100)	(311)	(211)	(331)	(511)
{C}_{hkl} \, (\AA)	1.025	0.676	0.289	0.285	0.149	0.121	0.047

**Table:** 

Space group	*P*3_2_21 (No. 154)
Laue class	\bar 3m
Number of atoms in the unit cell	9
Lattice parameters (Å)	a = 4.91, c = 5.40

**Table:** Asymmetric unit

Atom name	Charge	Position	Wyckoff letter and multiplicity	Local symmetry
Si	14	[0.47 00]	3*a*	2
O	8	[0.41 0.27 0.12]	6*b*	1

**Table 5:** Magnitudes of *C*_*hkl*_ in quartz

(*hkl*)	(011)	(101)	(203)	(112)	(031)	(110)	(010)	(102)
{C}_{hkl} \, (\AA)	0.714	0.483	0.213	0.209	0.170	0.165	0.138	0.114

**Table:** 

Space group	*R*3*c* (No. 161)
Laue class	\bar 3m
Number of atoms per unit cell	30
Lattice parameters (Å)	a = 5.15160\,(10)\, c = 13.8690\,(6)

**Table:** Asymmetric unit

Atom name	Charge	Position	Wyckoff	Local symmetry
Li	3	[00 0.3014]	6*a*	3
Nb	41	[00 0.0199]	6*a*	3
O	8	\left[0.0488\,\,\, 0.3438\,\,\, {{1}\over{12}}\right]	18*b*	1

**Table 7:** Magnitudes of *C*_*hkl*_ in LiNbO_3_

(*hkl*)	(012)	(104)	(110)	(001)	(101)	(116)	(122)	(010)
{C}_{hkl}\, (\AA)	1.556	0.945	0.776	0.572	0.562	0.482	0.392	0.390

**Table 8:** Magnitudes of *C*_*hkl*_ in AlN [wurzite structural type, space group type *P*6_3_ *mc*, the structure is reported by Xu & Ching (1993 ▸)]

(*hkl*)	(001)	(010)	(1\bar 20)	(011)	(013)	(\bar 122)	(012)	(021)
{C}_{hkl} \,(\AA)	0.774	0.654	0.462	0.385	0.257	0.227	0.189	0.100

**Table 9:** Magnitudes of *C*_*hkl*_ in CaF_2_ [fluorite structural type, space group type Fm\bar 3m, the structure is reported by Speziale & Duffy, 2002 ▸)]

(*hkl*)	(110)	(111)	(100)	(311)	(211)	(331)	(511)	(310)
{C}_{hkl}\, (\AA)	0.718	0.648	0.338	0.285	0.182	0.141	0.067	0.040

**Table 10:** Magnitudes of *C*_*hkl*_ in diamond/C (space group type Fm\bar 3m)

(*hkl*)	(111)	(110)	(311)	(200)	All the rest
{C}_{hkl}\, (\AA)	0.465	0.222	0.045	0.016	0

**Table 11:** Magnitudes of *C*_*hkl*_ in pyrite [FeS_2_, space group type Fm\bar 3m, the structure is reported by Ramsdell (1925 ▸)]

(*hkl*)	(011)	(113)	(111)	(102)	(001)	(012)	(112)	(115)
{C}_{hkl}\, (\AA)	0.378	0.299	0.249	0.154	0.151	0.128	0.057	0.040

**Table 12:** Magnitudes of *C*_*hkl*_ in corundum [space group type R\bar 3c, the structure is reported by Lewis *et al.* (1982 ▸)]

(*hkl*)	(012)	(\bar 114)	(010)	(1\bar 20)	(1\bar 2\bar 6)	(2\bar 3\bar 4)	(0\, \overline{1} \,10)	(\bar 1\bar 13)
{C}_{hkl}\, (\AA)	0.520	0.406	0.314	0.260	0.259	0.131	0.130	0.072

**Table 13:** Cleavage energies of silicon (obtained from DFT calculations/experiment) against the length of the structural gaps calculated in this work

	(111)	(110)
Cleavage-energy DFT (Pérez & Gumbsch, 2000 ▸) ({J/{m^2}})	2.88	3.46
Cleavage-energy experiment (Gleizer *et al.*, 2014 ▸) ({J /{m^2}})	2.2 ± 0.2	2.7 ± 0.3
Gap length (this work) (Å)	1.025	0.672

**Table 14:** Cleavage energies in LiNbO_3_ (obtained from an experiment) against the length of the structural gaps calculated in this work

	(012)	(010)
Cleavage-energy experiment (Hirsh *et al.*, 2020 ▸) ({J/{m^2}})	2.2	1.3
Gap length (this work) (Å)	1.55	0.39
