# Supplementary material for: Geometrical prediction of cleavage planes in crystal structures
Source: IUCrJ. 2021 Aug 20;8(Pt 5):793–804. doi: 10.1107/S2052252521007272 (PMC8420770; doi:10.1107/S2052252521007272)
Supplement: Supplementary file 2 [file m-08-00793-sup2.pdf]

GAps LOcations in Crystal Structures (GALOCS).

Release 01 (July 2021)

U. Vaknin, D. Sherman, S. Gorfman

Department of Materials Science and Engineering, Tel Aviv University, Israel

The algorithm for automatic searching of structural gaps in arbitrary crystal structures is described in the paper *"Geometrical prediction of cleavage planes in crystal structures"* (currently under review in the IUCrJ).

The program runs from MATLAB. Standalone versions will be available in the next releases.

1. Unpacking

- Extract all the content of the GALOCS.zip folder to any convenient location (e.g. c:\GALOCS). The unpacked content will include
  - **CIF_Library**. This folder contains CIF files for the calculations. Several CIF files are already stored there. The users can add more files (e.g. download from the Crystallography Open Database).
  - **Results.** This folder is used by the program to store the results of the calculations.
  - **PrimitiveCellSettings:** This folder contains the transformation matrices to the primitive unit cells.
  - **V10.** This folder contains all the MATLAB scripts
  - **Working examples.** The examples scripts for the calculations for several crystal structures.

- Add the folder V10 to the MATLAB path. This is done by running the command ***addpath V10*** from the root folder where the package was unpacked (e.g. c:\GALOCS). Alternatively add the line ***addpath c:\GALOCS\V10*** (use the actual folder name instead of c:\GALOCS) to the MATLAB startup file.

2. Running the program

- Go to the root folder.
- Define the CIF file. For example type **CIF = 'CIF_Library\Si.cif'**.

- Run **GammaList_ED(CIF,dmin,Tprimitive,NameSave)**. The input parameters include "dmin" (the smallest interplanar distance), "Tprimitive" (the matrix of transformation to the primitive unit cell) and "NameSave" (the name of the variable where the results are saved).
- Run ***[ResFull, ResUnique] = GapCleavageAbilityListFromData(NameLoad,y,NameSave)***. NameLoad is the file name from which the results of the previous calculations were loaded. "y" is the parameter defining the gap (see the paper and also y = 0.75). NameSave is the file name where the results are saved.
- The result of the calculations is stored in the variables **ResFull** (full list) and **ResUnique** (only symmetry independent planes). Both have format $h\ k\ l\ C_{hkl}$ (per row).
- It is now possible to inspect the $\Gamma(hkl, D)$ curves by calling the script **InspectGammaCurve(hkl,NameResultsGamma)**

3. Example 1. Silicon $(Fd\bar{3}m)$

Run the following script from the root folder.

ResUnique = Example1_Silicon

This should produce the following output in the variable ResUnique:

```
   -1.0000   -1.0000   -1.0000    1.0304
   -2.0000   -2.0000         0    0.6812
   -2.0000         0         0    0.2974
   -3.0000   -1.0000   -1.0000    0.2878
   -4.0000   -2.0000   -2.0000    0.1515
   -3.0000   -3.0000   -1.0000    0.1225
   -5.0000   -1.0000   -1.0000    0.0482
   -6.0000   -2.0000         0    0.0170
   -5.0000   -3.0000   -1.0000    0.0065
   -8.0000   -6.0000   -4.0000         0
   -9.0000   -5.0000   -3.0000         0
   -7.0000   -7.0000   -3.0000         0
   -8.0000   -6.0000   -2.0000         0
   -9.0000   -5.0000   -1.0000         0
  -10.0000   -4.0000         0         0
   -7.0000   -7.0000   -1.0000         0
```

To inspect the $\Gamma(hkl, D)$ curve call the function (replace [1,1,1] by any other [h,k,l])

InspectGammaCurve([1,1,1],'SiGamma.mat')

4. **Example 2. Quartz** $(P3_221)$

ResUnique = Example2_Quartz

This should produce the following output in the variable ResUnique:

```
        0   -1.0000   -1.0000    0.7140
   1.0000   -1.0000   -1.0000    0.4831
   2.0000   -2.0000   -3.0000    0.2131
   1.0000   -2.0000   -2.0000    0.2092
        0   -3.0000   -1.0000    0.1701
   1.0000   -2.0000         0    0.1650
        0   -1.0000         0    0.1376
   1.0000   -1.0000   -2.0000    0.1139
   1.0000   -3.0000   -1.0000    0.0881
   1.0000   -3.0000   -2.0000    0.0778
   3.0000   -3.0000   -2.0000    0.0763
   1.0000   -1.0000   -4.0000    0.0743
   2.0000   -3.0000   -1.0000    0.0717
   1.0000   -4.0000         0    0.0505
   2.0000   -3.0000   -3.0000    0.0501
   1.0000   -2.0000   -4.0000    0.0433
   1.0000   -2.0000   -1.0000    0.0358
   2.0000   -2.0000   -1.0000    0.0342
        0   -2.0000   -3.0000    0.0334
        0   -1.0000   -5.0000    0.0322
   1.0000   -4.0000   -2.0000    0.0299
   1.0000   -2.0000   -3.0000    0.0275
        0   -3.0000   -2.0000    0.0272
   4.0000   -4.0000   -1.0000    0.0234
   2.0000   -3.0000   -2.0000    0.0144
   2.0000   -3.0000   -4.0000    0.0099
        0   -1.0000   -3.0000    0.0087
   1.0000   -4.0000   -1.0000    0.0086
        0         0   -1.0000    0.0062
   3.0000   -4.0000   -2.0000    0.0052
   2.0000   -4.0000   -3.0000    0.0051
   2.0000   -4.0000   -1.0000    0.0031
   1.0000   -3.0000         0    0.0002
   1.0000   -1.0000   -5.0000         0
   1.0000   -3.0000   -4.0000         0
        0   -1.0000   -4.0000         0
```

Example 3. $LiNbO_3$ $(R3c)$

ResUnique = Example3_LiNbO3_R3c

This should produce the following output in the variable ResUnique:

```
    1.0000         0   -2.0000    1.5561
         0    1.0000   -4.0000    0.9446
    1.0000    1.0000         0    0.7660
         0         0   -3.0000    0.5704
         0    1.0000   -1.0000    0.5622
    1.0000    1.0000   -6.0000    0.4824
    2.0000    1.0000   -2.0000    0.3918
         0    3.0000         0    0.3896
    1.0000         0   -8.0000    0.3743
    1.0000    2.0000   -4.0000    0.3371
         0    1.0000  -10.0000    0.2459
    1.0000    3.0000   -2.0000    0.2053
    1.0000    1.0000   -3.0000    0.1888
    2.0000    1.0000   -8.0000    0.1795
         0    3.0000   -6.0000    0.1604
    1.0000         0   -5.0000    0.1574
    3.0000    1.0000   -4.0000    0.1443
    2.0000         0   -1.0000    0.1422
    1.0000    2.0000  -10.0000    0.1156
    1.0000    1.0000  -12.0000    0.1090
    1.0000    3.0000   -8.0000    0.0839
    3.0000    2.0000   -2.0000    0.0753
         0    1.0000   -7.0000    0.0460
         0    2.0000   -5.0000    0.0319
    1.0000    2.0000   -1.0000    0.0225
         0    1.0000  -13.0000         0
    2.0000    1.0000  -11.0000         0
         0    2.0000  -11.0000         0
         0    3.0000   -9.0000         0
    1.0000         0  -11.0000         0
```

5. Example 4. $LiNbO_3$ $(R\bar{3}c)$

ResUnique = Example4_LiNbO3_Rm3c

This should produce the following output in the variable ResUnique:

```
    1.0000         0   -2.0000    1.4369
         0    1.0000   -4.0000    0.8307
```

0	0	-3.0000	0.6132
1.0000	1.0000	0	0.6101
1.0000	1.0000	-6.0000	0.4577
0	3.0000	0	0.4086
0	1.0000	-1.0000	0.4039
2.0000	1.0000	-2.0000	0.3487
1.0000	0	-8.0000	0.2920
1.0000	2.0000	-4.0000	0.2836
0	1.0000	-10.0000	0.2150
1.0000	3.0000	-2.0000	0.2047
2.0000	1.0000	-8.0000	0.1510
1.0000	1.0000	-3.0000	0.1412
2.0000	0	-1.0000	0.1356
0	3.0000	-6.0000	0.1119
3.0000	1.0000	-4.0000	0.1083
1.0000	2.0000	-10.0000	0.0973
1.0000	0	-5.0000	0.0954
2.0000	3.0000	-4.0000	0.0924
1.0000	1.0000	-12.0000	0.0646
1.0000	3.0000	-8.0000	0.0577
3.0000	2.0000	-2.0000	0.0537
0	2.0000	-5.0000	0.0313
1.0000	2.0000	-1.0000	0.0254
0	1.0000	-7.0000	0.0213
0	1.0000	-13.0000	0
2.0000	1.0000	-11.0000	0
0	2.0000	-11.0000	0
2.0000	2.0000	-9.0000	0
0	3.0000	-9.0000	0

6. Example 5. AlN $(P6_3mc)$

ResUnique = Example5_AlN

This should produce the following output in the variable ResUnique:

0	0	-1.0000	0.7744
0	-1.0000	0	0.6540
1.0000	-2.0000	0	0.4620
0	-1.0000	-1.0000	0.3857
0	-1.0000	-3.0000	0.2569
1.0000	-2.0000	-2.0000	0.2265
0	-1.0000	-2.0000	0.1894

0	-2.0000	-1.0000	0.1004
0	-2.0000	-3.0000	0.0868
0	-1.0000	-5.0000	0.0448
1.0000	-2.0000	-4.0000	0.0355
1.0000	-3.0000	-1.0000	0.0272
1.0000	-3.0000	-3.0000	0.0155
0	-3.0000	-2.0000	0.0138
1.0000	-3.0000	0	0.0041
0	-2.0000	-5.0000	0.0004
1.0000	-2.0000	-5.0000	0
0	-1.0000	-4.0000	0
1.0000	-2.0000	-3.0000	0
1.0000	-3.0000	-2.0000	0
0	-3.0000	-1.0000	0
1.0000	-2.0000	-1.0000	0

7. **Example 6. CaF2** $(Fd\bar{3}m)$

   ResUnique = Example6_Fluorite

This should produce the following output in the variable ResUnique:

-2.0000	-2.0000	0	0.7189
-1.0000	-1.0000	-1.0000	0.6475
-2.0000	0	0	0.3379
-3.0000	-1.0000	-1.0000	0.2852
-4.0000	-2.0000	-2.0000	0.1817
-3.0000	-3.0000	-1.0000	0.1413
-5.0000	-1.0000	-1.0000	0.0666
-6.0000	-2.0000	0	0.0396
-5.0000	-3.0000	-1.0000	0.0271
-4.0000	-2.0000	0	0.0236
-4.0000	-4.0000	-2.0000	0.0051
-5.0000	-3.0000	-3.0000	0.0048
-5.0000	-5.0000	-3.0000	0
-6.0000	-4.0000	-2.0000	0
-7.0000	-3.0000	-1.0000	0
-5.0000	-5.0000	-1.0000	0
-6.0000	-4.0000	0	0
-7.0000	-1.0000	-1.0000	0

8. **Example 7. SrTiO3** $(Pm\bar{3}m)$

ResUnique = Example7_SrTiO3

This should produce the following output in the variable ResUnique:

```
        0   -1.0000   -1.0000    1.0554
        0         0   -1.0000    0.7428
  -1.0000   -1.0000   -2.0000    0.3691
  -1.0000   -1.0000   -1.0000    0.2214
        0   -1.0000   -3.0000    0.1944
  -1.0000   -2.0000   -3.0000    0.1069
        0   -1.0000   -2.0000    0.0562
  -1.0000   -1.0000   -4.0000    0.0527
  -1.0000   -1.0000   -3.0000    0.0486
  -2.0000   -3.0000   -3.0000    0.0172
  -1.0000   -3.0000   -3.0000    0.0059
        0   -2.0000   -3.0000    0.0054
  -2.0000   -2.0000   -3.0000    0.0017
        0   -1.0000   -4.0000    0.0017
  -1.0000   -2.0000   -5.0000         0
        0   -2.0000   -5.0000         0
  -1.0000   -1.0000   -5.0000         0
        0   -1.0000   -5.0000         0
  -2.0000   -3.0000   -4.0000         0
```

9. **Example 8. Diamond** $(Fm\bar{3}m)$

ResUnique = Example8_Diamond

This should produce the following output in the variable ResUnique:

```
  -1.0000   -1.0000   -1.0000    0.4649
  -2.0000   -2.0000         0    0.2216
  -3.0000   -1.0000   -1.0000    0.0445
  -2.0000         0         0    0.0155
  -7.0000   -5.0000   -1.0000         0
  -6.0000   -4.0000   -4.0000         0
  -7.0000   -3.0000   -3.0000         0
  -8.0000   -2.0000   -2.0000         0
  -5.0000   -5.0000   -3.0000         0
  -6.0000   -4.0000   -2.0000         0
  -7.0000   -3.0000   -1.0000         0
  -8.0000   -2.0000         0         0
  -5.0000   -5.0000   -1.0000         0
```

-6.0000	-4.0000	0	0
-5.0000	-3.0000	-3.0000	0
-7.0000	-1.0000	-1.0000	0
-4.0000	-4.0000	-2.0000	0
-5.0000	-3.0000	-1.0000	0
-6.0000	-2.0000	0	0
-4.0000	-2.0000	-2.0000	0
-5.0000	-1.0000	-1.0000	0
-3.0000	-3.0000	-1.0000	0
-4.0000	-2.0000	0	0

10. Example 9. Pyrite $(Pa3)$

ResUnique = Example9_Pyrite

0	-1.0000	-1.0000	0.3784
-1.0000	-1.0000	-3.0000	0.2990
-1.0000	-1.0000	-1.0000	0.2487
-1.0000	0	-2.0000	0.1543
0	0	-1.0000	0.1508
0	-1.0000	-2.0000	0.1276
-1.0000	-1.0000	-2.0000	0.0568
-1.0000	-1.0000	-5.0000	0.0400
-1.0000	-3.0000	-3.0000	0.0268
-1.0000	-3.0000	-5.0000	0.0249
-3.0000	-1.0000	-5.0000	0.0249
-1.0000	-2.0000	-2.0000	0.0112
-1.0000	-2.0000	-3.0000	0.0078
0	-2.0000	-5.0000	0.0039
-1.0000	0	-4.0000	0.0007
-3.0000	-1.0000	-4.0000	0.0006
-1.0000	-2.0000	-6.0000	0
-2.0000	-1.0000	-6.0000	0
-1.0000	-1.0000	-6.0000	0
0	-1.0000	-6.0000	0
-1.0000	0	-6.0000	0
-2.0000	-4.0000	-5.0000	0
-1.0000	-4.0000	-5.0000	0

11. Example 10. Corundum $(R\bar{3}c)$

ResUnique = Example10_Corundum

1.0000	0	-2.0000	0.5200
0	1.0000	-4.0000	0.4064
0	3.0000	0	0.3138
1.0000	1.0000	0	0.2592
1.0000	1.0000	-6.0000	0.2586
1.0000	2.0000	-4.0000	0.1311
0	1.0000	-10.0000	0.1307
1.0000	1.0000	-3.0000	0.0719
1.0000	0	-5.0000	0.0602
1.0000	0	-8.0000	0.0590
1.0000	2.0000	-10.0000	0.0389
0	1.0000	-1.0000	0.0361
0	0	-3.0000	0.0280
3.0000	1.0000	-4.0000	0.0211
2.0000	1.0000	-2.0000	0.0150
2.0000	3.0000	-4.0000	0.0138
3.0000	1.0000	-10.0000	0.0095
1.0000	1.0000	-9.0000	0.0077
1.0000	4.0000	0	0.0066
1.0000	0	-14.0000	0.0055
2.0000	2.0000	-3.0000	0.0022
0	1.0000	-7.0000	0.0020
0	2.0000	-5.0000	0.0009
1.0000	1.0000	-15.0000	0
1.0000	2.0000	-13.0000	0
